# Mutational spectrum and prognosis in Chinese patients with prefibrotic primary myelofibrosis

**DOI:** 10.1002/jha2.361

**Published:** 2021-12-30

**Authors:** Chi‐Keung Cheng, Jennifer W. Y. Lai, Yuk‐Lin Yung, Hoi‐Yun Chan, Raymond S. M. Wong, Natalie P. H. Chan, Joyce S. Cheung, Xi Luo, Herbert‐Augustus Pitts, Margaret H. L. Ng

**Affiliations:** ^1^ Blood Cancer Cytogenetics and Genomics Laboratory Department of Anatomical and Cellular Pathology Prince of Wales Hospital The Chinese University of Hong Kong Hong Kong China; ^2^ Department of Medicine and Therapeutics, Prince of Wales Hospital The Chinese University of Hong Kong Hong Kong China; ^3^ Sir Y. K. Pao Centre for Cancer, Prince of Wales Hospital Hong Kong China; ^4^ State Key Laboratory of Translational Oncology The Chinese University of Hong Kong Hong Kong China

**Keywords:** myeloproliferative neoplasms, prefibrotic primary myelofibrosis, prognostic factors, RUNX1, TP53

## Abstract

Prefibrotic primary myelofibrosis (Pre‐PMF) has been classified as a separate entity of myeloproliferative neoplasms (MPNs). Pre‐PMF is clinically heterogeneous but a specific prognostic model is lacking. Gene mutations have emerged as useful tools for stratification of myelofibrosis patients. However, there have been limited studies comprehensively investigating the mutational spectrum and its clinicopathological significance in pre‐PMF subjects. In this study, we addressed these issues by profiling the mutation status of 141 genes in 172 Chinese MPN patients including 72 pre‐PMF cases. Our findings corroborated the clinical/molecular distinctiveness of pre‐PMF and suggested a refined risk classification strategy for this entity.

## 1 INTRODUCTION

The recent World Health Organization (WHO) classification has divided primary myelofibrosis (PMF) into prefibrotic/early and overt PMF [1]. The clinical severity of prefibrotic PMF (pre‐PMF) varies markedly from isolated thrombocytosis to symptoms of high‐risk PMF [2]. The International Prognostic Scoring System (IPSS) has been insufficient to stratify this group of patients [3]. A specific prognostic schema for this entity is yet to be established. The high mutation risk (HMR) status (defined by the presence of at least 1 mutated gene including *ASXL1*, *SRSF2*, *EZH2*, and *IDH1/2*) [4] has been demonstrated as a significant outcome predictor in pre‐PMF [3]. Nonetheless, as there is emerging evidence implicating different pathogenesis of PMF in Chinese subjects [5, 6, 7, 8], the prevalence and prognostic significance of these HMR and/or other mutations in Chinese pre‐PMF patients remain to be determined. Here, we investigated the mutational landscape in 72 Chinese patients with pre‐PMF, characterized their differences from closely related myeloproliferative neoplasms (MPNs), and suggested a better risk stratification approach.

## 2 PATIENTS AND METHODS

We reviewed and recruited Chinese patients with essential thrombocythemia (ET) and PMF between 2005 and 2020 in our hospital fulfilling the 2016 WHO criteria [1] and with available diagnostic DNA sample for molecular testing. Cases with equivocal bone marrow morphological findings had been excluded. The retrieved cohort included 72 ET, 72 pre‐PMF, and 28 overt PMF cases. Informed consent was obtained from all patients, and the study was approved by the Joint Chinese University of Hong Kong‐New Territories East Cluster Clinical Research Ethics Committee and followed the Declaration of Helsinki. Of the 72 pre‐PMF patients (M:F = 45:27; median age = 65 years) (Table 1), 64 (89%) received treatment (hydroxyurea alone [*n *= 53], anagrelide alone [*n *= 2], hydroxyurea/anagrelide [*n *= 5], hydroxyurea/anagrelide/interferon [*n *= 1], hydroxyurea/thalidomide/prednisone [*n *= 1], interferon/anagrelide [*n *= 1], and ruxolitinib [*n *= 1]). None of the patients underwent allogeneic stem cell transplantation.

**TABLE 1:** Characteristics of the Chinese patients with WHO‐defined pre‐PMF, overt PMF, and ET

Variables*	ET (*n *= 72)	Pre‐PMF (*n *= 72)	Overt PMF (*n *= 28)	*p* Value (ET vs. Pre‐PMF)	*p* Value (Pre‐PMF vs. overt PMF)
Age, median (range)	61 (21–81)	65 (30–86)	64 (45–85)	**0.040**	0.682
Age > 65 years, *n* (%)	22 (31%)	33 (46%)	14 (50%)	0.086	0.824
Male sex, *n* (%)	29 (40%)	45 (63%)	17 (61%)	**0.012**	1.000
Hemoglobin, g/dl, median (range)	13.5 (10–16.6)	13.1 (7.3–15.9)	8.5 (4.8–13.2)	0.075	**<0.0001**
Hemoglobin < 10 g/dl, *n* (%)	0 (0%)	7 (10%)	20 (71%)	**0.013**	**<0.0001**
Leukocytes, × 10^9^/L, median (range)	8.8 (4.9–17.4)	14.4 (5.5–96.4)	8.4 (0.7–58.9)	**<0.0001**	**0.023**
Leukocytes > 25 × 10^9^/L, *n* (%)	0 (0%)	7 (10%)	3 (11%)	**0.013**	1.000
Leukocytes < 4 × 10^9^/L, *n* (%)	0 (0%)	0 (0%)	6 (21%)	1.000	**0.0003**
Platelets, × 10^9^/L, median (range)	731 (451–1989)	742 (131–2147)	254 (8–1160)	0.857	**<0.0001**
Platelets < 100 × 10^9^/L, *n* (%)	0 (0%)	0 (0%)	9 (32%)	1.000	**<0.0001**
LDH, U/L, median (range)	221 (128–605)	290 (93–705)	607 (160–1346)	**<0.0001**	**<0.0001**
LDH > normal range, *n* (%)	41 (67%)	56 (78%)	27 (96%)	**0.012**	**0.035**
Circulating blasts, %, median (range)	0 (0–0)	0 (0–1)	1 (0–7)	0.159	**<0.0001**
Circulating blasts ≥1%, *n* (%)	0 (0%)	1 (1%)	18 (64%)	1.000	**<0.0001**
Constitutional symptoms, *n* (%)	2 (3%)	12 (17%)	16 (57%)	**0.009**	**0.0001**
Palpable splenomegaly, *n* (%)	0 (0%)	22 (31%)	26 (93%)	**<0.0001**	**<0.0001**
Leukemic transformation, *n* (%)	0 (0%)	4 (6%)	3 (11%)	0.120	0.396
Death, *n* (%)	5 (7%)	19 (26%)	18 (64%)	**0.003**	**0.001**
Follow‐up time, months, median (range)	28 (3–175)	46 (4–118)	39 (2–118)	0.124	**0.024**
***Cytogenetics, n* (%)**				**/**	**0.009**
Favorable	/	49 (98%)	16 (80%)		
Unfavorable	/	1 (2%)	1 (5%)		
Very high risk	/	0 (0%)	3 (15%)		
Unknown	/	22	8		
***IPSS risk stratification, n* (%)**				**/**	**<0.0001**
Low	/	34 (47%)	1 (4%)		
Intermediate‐1	/	24 (33%)	6 (21%)		
Intermediate‐2	/	9 (13%)	6 (21%)		
High	/	5 (7%)	15 (54%)		
***Driver types, n* (%)**				0.272	0.054
*JAK2* V617F	51 (71%)	56 (78%)	16 (57%)		
*CALR* type 1‐like	4 (6%)	7 (10%)	7 (25%)		
*CALR* type 2‐like	3 (4%)	0 (0%)	1 (4%)		
*CALR* nontype 1/2‐like	2 (3%)	0 (0%)	1 (4%)		
*MPL* W515	4 (6%)	2 (3%)	1 (4%)		
Triple negative	8 (11%)	7 (10%)	2 (7%)		
***Allele burden, median (range)***					
*JAK2* V617F	0.25 (0.05–0.70)	0.41 (0.11–0.98)	0.48 (0.06–0.89)	**<0.0001**	0.430
*CALR*	0.42 (0.07–0.47)	0.46 (0.31–0.55)	0.46 (0.45–0.82)	**0.025**	0.604
*MPL* W515	0.19 (0.14–0.30)	0.49 (0.30–0.68)	0.75 (0.75–0.75)	0.200	0.667
***Nondriver mutations, n* (%)**					
*TET2*	11 (15%)	13 (18%)	2 (7%)	0.824	0.223
*ASXL1*	2 (3%)	12 (17%)	8 (29%)	**0.009**	0.264
*SRSF2*	1 (1%)	5 (7%)	2 (7%)	0.209	1.000
*DNMT3A*	8 (11%)	4 (6%)	2 (7%)	0.367	0.671
*NF1*	0 (0%)	4 (6%)	1 (4%)	0.120	1.000
*ZRSR2*	0 (0%)	4 (6%)	3 (11%)	0.120	0.396
*RUNX1*	0 (0%)	3 (4%)	1 (4%)	0.245	1.000
*BCOR*	1 (1%)	2 (3%)	0 (0%)	1.000	0.484
*FBXW7*	0 (0%)	2 (3%)	0 (0%)	0.497	0.484
*PDGFRA*	0 (0%)	2 (3%)	0 (0%)	0.497	0.484
*PMS2*	0 (0%)	2 (3%)	2 (7%)	0.497	0.312
*STAG2*	0 (0%)	2 (3%)	0 (0%)	0.497	0.484
*TP53*	0 (0%)	2 (3%)	0 (0%)	0.497	0.484
***HMR, n* (%)**				**0.007**	0.510
0	69 (96%)	58 (81%)	20 (71%)		
1 mutated gene	3 (4%)	10 (14%)	5 (18%)		
≥2 mutated genes	0 (0%)	4 (6%)	3 (11%)		
No. of nondriver mutations, median (range)	1 (0–4)	1 (0–8)	1 (0–5)	0.185	**0.048**

LDH, lactate dehydrogenase.

* Clinical/laboratory findings were obtained at diagnosis. Cytogenetics was classified according to the revised cytogenetic stratification in PMF [16] and the percentages were calculated from the available cases. *CALR* mutations were classified as previously described [17]. For nondriver mutations, only genes recurrently mutated (> 1 patient) in the pre‐PMF cases are shown. HMR genes include *ASXL1*, *SRSF2*, *EZH2*, and *IDH1/2*.

*Note*: Significant *p* values (< 0.05) are in bold.

Mutational profiling of 141 genes (Table S1) was performed by targeted next‐generation sequencing (NGS). Libraries were prepared from diagnostic DNA from 151 bone marrow and 21 peripheral blood samples using the QIAseq Targeted Human Myeloid Neoplasms Panel (Qiagen) and sequenced on the Illumina NextSeq 500. The median time from diagnosis to the date of NGS did not differ significantly between the ET (35.5 months), pre‐PMF (35.2 months), and overt PMF (52.1 months) groups. Identification of high‐confident somatic variants was performed as previously described [9] (Detailed in the Supporting information). Categorical and continuous variables between groups were analyzed by Fisher exact and Mann–Whitney tests, respectively. Overall survival (OS) was measured from the date of diagnosis to date of death or last follow‐up. Variables with *p *< 0.1 in univariate Cox regression analysis were included in multivariate analysis. Two‐sided *p *< 0.05 were considered statistically significant.

## 3 RESULTS

**FIGURE 1:**
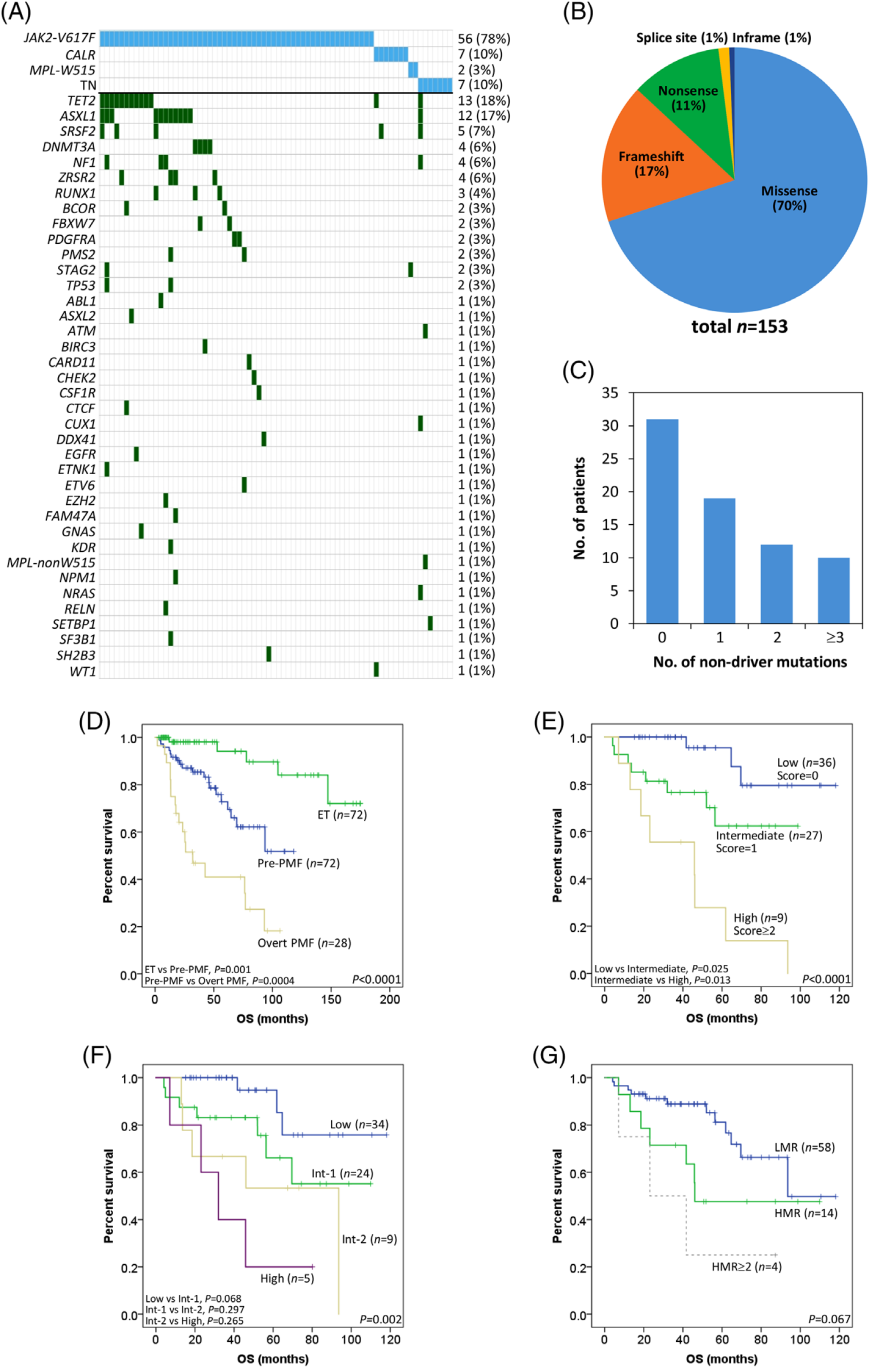
Mutations and prognosis in Chinese pre‐PMF patients. (A) A waterfall plot showing the distribution of mutations in the pre‐PMF patients (*n *= 72). Genes mutated in the cohort are shown on the left of the plot, while the number and percentage of the patients with the gene mutations are shown on the right. TN, triple negative genotype. (B) A pie chart showing the proportion of different types of the mutations identified in the pre‐PMF patients. (C) Nondriver mutational burden in the pre‐PMF patients. Approximately 43%, 26%, 17%, and 14% of the patients had 0, 1, 2, and ≥ 3 nondriver mutations, respectively. (D) OS of patients with pre‐PMF, overt PMF and ET recruited in this study. Three patients with overt PMF received allogeneic hematopoietic stem cell transplant and their survival data had been censored at the time of transplantation. (E) Risk stratification of pre‐PMF patients according to the new 4‐factor scoring system. Patients were stratified into three risk groups (low, intermediate, and high) based on their risk scores. (F) Risk stratification of pre‐PMF patients according to the IPSS system. Although overall the curves were significantly different, there were insufficient distinction between adjacent risk groups. Int‐1 and Int‐2 represent intermediate‐1 and intermediate‐2, respectively. (G) Risk stratification of pre‐PMF patients according to the HMR mutational status. A low mutation risk (LMR) and high mutation risk (HMR) status is defined by the absence or presence of at least 1 mutated HMR gene (*ASXL1*, *SRSF2*, *EZH2*, and *IDH1/2*), respectively according to Guglielmelli et al. [3]. The survival curve of patients with ≥2 mutated HMR genes is also shown. In panel D–G, Kaplan–Meier survival curves were compared by log‐rank test

Of the 72 pre‐PMF patients studied, 19 patients died and 4 sustained leukemic transformations. The median follow‐up time was 46 months (4–118 months). The causes of death included bleeding (*n *= 7), transformation to AML (*n *= 4), infection (*n *= 3), second neoplasm (*n *= 2), cardiac arrest (*n *= 1), respiratory failure (*n *= 1), and unknown (*n *= 1). Twelve (17%) cases had no fibrosis in the bone marrow (MF‐0). Cytogenetic data were unavailable from 22 (31%) patients (21 had no karyotyping done and 1 had failed karyotyping). Of the 50 (69%) available cases, 49 (98%) were of favorable (including 43 normal karyotype) cytogenetics. A total of 153 mutations in 40 genes were identified in the pre‐PMF cohort (Figure 1A–C; Table S2), and the median variant allele frequency of all the mutations was 0.42. Mutually exclusive driver mutations of *JAK2* V617F, *CALR* (all type 1‐like), and *MPL* (W515) were detected in 56 (78%), 7 (10%), and 2 (3%) patients, respectively. Thirteen genes other than *JAK2*/*CALR*/*MPL* were recurrently mutated (>1 patient), among which *TET2*, *ASXL1*, *SRSF2*, *DNMT3A*, *NF1*, and *ZRSR2* were altered in >5% of the cases. The number and distribution of nondriver mutations did not vary significantly among the driver types (Table S3). Overall, each patient carried 0–8 nondriver changes, which increased with patients’ age (*r *= 0.403, *p *= 0.0005). *ASXL1* was found to co‐mutate with *NF1* (adjusted *p *= 0.039) after correction for multiple comparisons.

Anemia (hemoglobin < 10 g/dl) (*p *< 0.0001), leukopenia (leukocytes < 4 × 10^9^/L) (*p *= 0.0003), thrombocytopenia (platelets < 100 × 10^9^/L) (*p *< 0.0001), circulating blasts ≥ 1% (*p *< 0.0001), constitutional symptoms (*p *= 0.0001), splenomegaly (*p *< 0.0001) and nonfavorable cytogenetics (*p *= 0.009) were less common in pre‐PMF than overt PMF patients (Table 1). Accordingly, pre‐PMF subjects were enriched in the lower‐risk IPSS categories (80% vs. 25% of overt PMF, *p *< 0.0001). Lactate dehydrogenase (LDH) levels were also lower in pre‐PMF than overt PMF (*p *< 0.0001). On the other hand, pre‐PMF patients were older (*p *= 0.04) with higher leukocyte counts and LDH levels (both *p *< 0.0001), and more often had anemia (*p *= 0.013), constitutional symptoms (*p *= 0.009), and a palpable spleen (*p *< 0.0001) than ET subjects. Pre‐PMF also contrasted with ET in male predominance (*p *= 0.012). OS varied significantly among the three MPNs with pre‐PMF having an intermediate outcome (5‐year OS rate: 41% vs. 73% vs. 94%, *p *< 0.0001) (Figure 1D). Regarding genetic changes, although individual nondriver mutations were similarly distributed between the two PMF groups, the mutational burden was higher in overt PMF (*p *= 0.048). Compared with ET, pre‐PMF patients had higher *JAK2* V617F (*p *< 0.0001) and *CALR* (*p *= 0.025) mutant levels and more frequent *ASXL1* mutations (*p *= 0.009). HMR mutations were overall more common in pre‐PMF than ET (20% vs. 4%, *p *= 0.007) but no such difference was noted between pre‐PMF and overt PMF.

Among the 72 pre‐PMF patients, those with mutated *CALR* and the *JAK2/CALR/MPL* triple negative (TN) genotype were younger (*p *= 0.023) (Table S3). Leukocyte counts were highest in patients with the TN status (*p *= 0.008). Among the *JAK2*‐mutated cases, the V617F allele burden correlated positively with leukocyte counts (*r *= 0.54, *p *< 0.0001). Platelet counts were highest in *CALR*‐mutated cases (*p *= 0.015). Concerning nondriver mutations, *TET2* (*p *= 0.027) and *STAG2* (*p *= 0.049) were associated with older age, with the former also predominated in male subjects (27% vs. 4%, *p *= 0.024) (Table S4). Moreover, *TET2* (*p *= 0.009) and *NF1* (*p *= 0.042) correlated with higher leukocyte counts, and *SRSF2* with lower hemoglobin levels (*p *= 0.046). None of the driver and nondriver mutations was associated with constitutional symptoms and splenomegaly. There was no specific mutational signature for those subjects with leukemic transformations.

Univariate analysis indicated that age > 65 years (*p *= 0.007), leukocytes > 25 × 10^9^/L (*p *= 0.0001), *TET2* (*p *= 0.005), *TP53* (*p *= 0.001), and ≥2 mutated HMR genes (*p *= 0.019) were associated with shortened OS in the pre‐PMF patients (Table S5). Higher *JAK2* V617F allele burden tended to confer poorer OS but no statistical significance was reached (*p *= 0.102). In multivariate analysis, older age (hazard ratio [HR] = 3.3), elevated leukocyte counts (HR = 3.6), *RUNX1* (HR = 8.0), and *TP53* (HR = 8.2) mutations emerged as independent adverse factors for OS. To construct a pre‐PMF risk model, a weighted score of 1 was assigned to age > 65 years and leukocytes > 25 × 10^9^/L, whereas a score of 2 was assigned to *RUNX1* and *TP53* mutations according to the individual HR of the factors. The overall score in our cohort ranged 0–4. On this basis, a three‐category risk model was devised with low‐ (score = 0), intermediate‐ (score = 1), and high‐risk (score ≥ 2) patients representing 50%, 38%, and 13% of the cohort, respectively. Compared with the low‐risk group, the HR for death was 4.2 (95% confidence interval [CI] = 1.1–15.8) for the intermediate‐risk and 14.6 (95% CI = 3.8–55.4) for the high‐risk groups. The 5‐year OS rate was 96% in low‐risk, 62% in intermediate‐risk, and 28% in high‐risk patients (*p *< 0.0001) (Figure 1E).

Contrary to this superior risk stratification, there was insufficient distinction between adjacent risk groups when the IPSS system was used for prognostic classification (Figure 1F). Also, the performance of the HMR status in the stratification was suboptimal (Figure 1G). Notably, among the nine high‐risk pre‐PMF patients stratified by the current model, six were classified as low/intermediate risks according to the IPSS. Conversely, two of the five high‐risk IPSS patients fell to the intermediate‐risk group in the new system. Other prognostic scoring systems (DIPSS+, MIPSS70+, GIPSS, and MIPSS70+ version 2.0) [10] were inadequate in stratifying our pre‐PMF subjects into distinct risk groups (Figure S1).

## 4 DISCUSSION

The differential clinicopathological features revealed in our comparative study are consistent with the recognition of pre‐PMF as a distinct entity. Molecularly, higher driver mutant burdens and mutated *ASXL1* might help further distinguish pre‐PMF from ET, whereas overt PMF was typified by more nondriver alterations. Factors affecting pre‐PMF prognosis are incompletely understood, and previous prognostic models were developed primarily for overt PMF [2]. Here, we proposed a 4‐factor scoring system for refined risk classification of pre‐PMF patients. Besides advanced age and leukocytosis, our data revealed that *RUNX1* and *TP53* also adversely impacted pre‐PMF survival. These genes were mutated in 7% of our pre‐PMF cohort in a nonoverlapping manner and have also been associated with poor prognosis in other myeloid neoplasms [11, 12]. Importantly, previous studies have indicated the negative prognostic impacts of these mutations in myelofibrosis patients [9, 13, 14], and particularly, mutated *TP53* has been identified as the worst subgroup [9, 14]. If our findings could be validated, this scoring system will be a useful tool to determine the prognosis of Chinese patients with pre‐PMF and guide clinical management.

Here, we observed that the TN genotype was not associated with poorer survival in our pre‐PMF patients as previously reported [3]. Likewise, Gill et al. have shown that this genotype is not an adverse factor in Chinese patients with overt PMF [8]. Of the five HMR genes, only *ASXL1* and *SRSF2* were recurrently mutated in our pre‐PMF cases. When considered individually, *SRSF2* (*p *= 0.042) but not *ASXL1* (*p *= 0.127) was associated with shortened OS. Recent studies have suggested that mutated *ASXL1* confers a worse prognosis when associated with high‐risk genes including *SRSF2* and *EZH2* [14]. Accordingly, we observed that the presence of at least two mutated HMR genes (*ASXL1*/*SRSF2* [*n *= 3] and *ASXL1*/*EZH2* [*n *= 1]) was significantly associated with inferior OS in univariate but not multivariate analysis. We also observed that mutated *TET2* was a poor prognostic factor for OS in our pre‐PMF subjects in univariate analysis only, possibly related to its confounding associations with other adverse factors including older age and higher leukocyte counts. Of note, mutated *U2AF1*, a potential high‐risk marker found in 11% of Mayo Clinic pre‐PMF subjects [15], was absent in our pre‐PMF patients. In view of the limited follow‐up time and infrequent gene mutations, larger collaborative studies are warranted to confirm the present findings.

Due to missing cytogenetic data, the impact of cytogenetics on our pre‐PMF patients was not evaluated. Nonetheless, as nearly all the pre‐PMF cases carried a favorable karyotype, the prognostic value of cytogenetics is expected to be low. The high proportion of pre‐PMF in our cohort might be related to early bone marrow examination and the revised classification that had led to better distinction between pre‐PMF and ET [1].

In summary, we have revealed the mutational spectrum and its clinicopathological significance in Chinese pre‐PMF patients and suggested a specific prognostic model for this entity. Our findings highlight the distinctiveness of pre‐PMF, substantiating the need for accurate diagnosis and a separate stratification strategy to guide better disease management.

## AUTHOR CONTRIBUTIONS

CKC designed and performed research and wrote the paper; JWYL collected and analyzed clinicopathological data and wrote the paper; YLY, HYC, XL, and HAP performed research and analyzed data; RSMW, NPHC, and JSC collected, reviewed, and analyzed clinicopathological data; MHLN designed and coordinated research and advised on revision of the paper.

## CONFLICT OF INTEREST

The authors declare no conflict of interest.

## Supporting information

Supporting information

Supporting information
