# Supplementary material for: Mutational spectrum and prognosis in Chinese patients with prefibrotic primary myelofibrosis
Source: EJHaem. 2021 Dec 30;3(1):184–90. doi: 10.1002/jha2.361 (PMC9176118; doi:10.1002/jha2.361)
Supplement: Supplementary file 1 — Supporting information [file JHA2-3-184-s001.docx]

**Supporting information**

**Methods**

***Amplicon sequencing, data processing and variant filtering***

The read processing, alignment (hg19 as the reference), calling and annotation of single nucleotide variants/small indels were performed with the unique molecular identifier (UMI)-based caller smCounter2 run on the GeneGlobe (Qiagen).^1^ The mean read depth of the target regions across all samples was 1,071×. Identification of high-confident somatic variants was based on the filtering strategy described by Grinfeld *et al*.^2^ Briefly, only non-synonymous variants in coding regions and mutations affecting the conserved splice donor (+1/+2)/acceptor (-1/-2) sites were considered for the analyses. Variants with a population frequency of ≥0.1% in the 1000 Genomes Project (Phase 3), Genome Aggregation Database (v2.1.1) or dbSNP (Build 154) were removed. Variants that matched entries in the Catalogue Of Somatic Mutations In Cancer (COSMIC v92) describing somatically acquired mutations at the same genomic or amino-acid position were retained. Also, loss-of-function mutations (nonsense, frameshift or splice sites) in myeloid driver genes^2^ including *ASXL1*, *BCOR*, *CUX1*, *DNMT3A*, *TET2*, *EZH2*, *GATA2*, *RB1*, *RUNX1*, *SH2B3*, *NF1*, *TP53*, *CBL*, *KMT2C* and *ZRSR2* were kept. On the other hand, variants matching a COSMIC entry but were not reported as somatic and had a variant allele frequency (VAF) of >40% were removed. Also, single nucleotide variants that were not prevalent in population databases, did not match to an entry in the COSMIC and had a VAF of >40% were discarded to eliminate possible rare and private germline polymorphisms. On the contrary, single nucleotide variants that were not reported in any population database but were within 3 amino-acids of a somatic variant reported in the COSMIC were retained if their VAF was less than 40%. All filtered variants were visually checked with the Integrative Genomics Viewer (IGV).

*CALR* mutations were concomitantly evaluated in all the cases by fragment analysis. The identity of the mutations was determined by Sanger sequencing.

As our routine testing on a commercial myeloid DNA reference standard (Horizon) containing 22 variants in 19 genes with this method consistently identified variants with a VAF of 5%, this was chosen as a cut-off for variant filtering and reporting.

**References**

1. Xu C, Gu X, Padmanabhan R, Wu Z, Peng Q, DiCarlo J, et al. smCounter2: an accurate low-frequency variant caller for targeted sequencing data with unique molecular identifiers. *Bioinformatics*. 2019;35(8):1299-1309.
2. Grinfeld J, Nangalia J, Baxter EJ, Wedge DC, Angelopoulos N, Cantrill R, et al. Classification and Personalized Prognosis in Myeloproliferative Neoplasms. *N Engl J Med*. 2018;379(15):1416-30.

**Table S1.** The 141 genes covered in the panel.

| *ABL1* | *BRCA2* | *CTCF* | *FAS* | *JAK1* | *LUC7L2* | *NTRK3* | *PTPN11* | *SRP72* | *U2AF2* |
| --- | --- | --- | --- | --- | --- | --- | --- | --- | --- |
| *ADA* | *BRINP3* | *CUX1* | *FBXW7* | *JAK2* | *MAP2K1* | *OR13H1* | *RAD21* | *SRSF2* | *WAS* |
| *ANKRD26* | *C17orf97* | *DAXX* | *FLRT2* | *JAK3* | *MLH1* | *OR8B12* | *RB1* | *STAG2* | *WRN* |
| *ASXL1* | *CALR* | *DDX41* | *FLT3* | *KAT6A* | *MPL* | *P2RY2* | *RELN* | *STAT3* | *WT1* |
| *ASXL2* | *CARD11* | *DNM2* | *GATA1* | *KCNA4* | *MSH2* | *PAX5* | *RUNX1* | *STXBP2* | *XPO1* |
| *ATM* | *CBL* | *DNMT1* | *GATA2* | *KCNK13* | *MSH6* | *PCDHB1* | *SAXO2* | *SUZ12* | *ZRSR2* |
| *ATRX* | *CBLB* | *DNMT3A* | *GJB3* | *KDM6A* | *MYC* | *PDGFRA* | *SETBP1* | *TAL1* |  |
| *BCL6* | *CBLC* | *EED* | *GNAS* | *KDR* | *MYD88* | *PHF6* | *SF1* | *TERC* |  |
| *BCOR* | *CDKN2A* | *EGFR* | *HNRNPK* | *KIT* | *NBN* | *PML* | *SF3A1* | *TERT* |  |
| *BCORL1* | *CEBPA* | *ELANE* | *HRAS* | *KLHDC8B* | *NF1* | *PMS2* | *SF3B1* | *TET2* |  |
| *BCR* | *CHEK2* | *EP300* | *IDH1* | *KLHL6* | *NOTCH1* | *PRAMEF2* | *SH2B3* | *TNFRSF13B* |  |
| *BIRC3* | *CREBBP* | *ETNK1* | *IDH2* | *KMT2A* | *NPAT* | *PRF1* | *SH2D1A* | *TP53* |  |
| *BLM* | *CRLF2* | *ETV6* | *IKZF1* | *KMT2C* | *NPM1* | *PRPF40B* | *SMARCB1* | *TPMT* |  |
| *BRAF* | *CSF1R* | *EZH2* | *IKZF3* | *KRAS* | *NRAS* | *PRPF8* | *SMC1A* | *TUBA3C* |  |
| *BRCA1* | *CSF3R* | *FAM47A* | *IL7R* | *LRRC4* | *NSD1* | *PTEN* | *SMC3* | *U2AF1* |  |

**Table S2.** The complete list of the mutations identified in 172 Chinese patients with WHO-defined pre-PMF, overt PMF and ET.

See separate Excel file**.**

**Table S3.** Characteristics of the Chinese pre-PMF patients according to the driver mutation types.

| **Variables^*^** | ***JAK2* V617F (*n*=56)** | ***CALR***  **(*n*=7)** | ***MPL* W515**  **(*n*=2)** | **TN**  **(*n*=7)** | ***P* value^#^** |
| --- | --- | --- | --- | --- | --- |
| Age, median (range) | 66 (30-86) | 58 (33-67) | 78 (75-80) | 57 (41-72) | **0.023** |
| Male sex, *n* (%) | 35 (63%) | 6 (86%) | 1 (50%) | 3 (43%) | 0.409 |
| Haemoglobin, g/dL, median (range) | 13.2 (8.0-15.9) | 13.5 (11.0-14.7) | 10.7 (10.0-11.3) | 12.6 (7.3-15.5) | 0.288 |
| Leukocytes, ×10^9^/L, median (range) | 14.8 (6.0-54.2) | 8.8 (6.9-16.1) | 6.8 (5.5-8.1) | 15.7 (8.4-96.4) | **0.008** |
| Platelets, ×10^9^/L, median (range) | 761 (131-2147) | 1065 (620-1640) | 624 (520-728) | 527 (392-980) | **0.015** |
| LDH, U/L, median (range) | 273 (93-705) | 320 (225-671) | 347 (256-437) | 316 (176-393) | 0.483 |
| Constitutional symptoms, *n* (%) | 10 (18%) | 0 (0%) | 0 (0%) | 2 (29%) | 0.557 |
| Palpable splenomegaly, *n* (%) | 17 (30%) | 3 (43%) | 0 (0%) | 2 (29%) | 0.899 |
| Leukemic transformation, *n* (%)  Death, n (%)  Follow-up time, months, median (range) | 4 (7%) | 0 (0%) | 0 (0%) | 0 (0%) | 1.000 |
| Death, *n* (%) | 18 (32%) | 0 (0%) | 0 (0%) | 1 (14%) | 0.315 |
| Follow-up time, months, median (range) | 47 (4-118) | 36 (19-93) | 51 (46-56) | 32 (18-89) | 0.907 |
|  |  |  |  |  |  |
| ***IPSS risk stratification, n (%)*** |  |  |  |  | 0.533 |
| Low / Intermediate-1 | 44 (79%) | 7 (100%) | 2 (100%) | 5 (71%) |  |
| Intermediate-2 / High | 12 (21%) | 0 (0%) | 0 (0%) | 2 (29%) |  |
|  |  |  |  |  |  |
| ***Non-driver mutations, n (%)*** |  |  |  |  |  |
| *TET2* | 11 (20%) | 1 (14%) | 0 (0%) | 1 (14%) | 1.000 |
| *ASXL1* | 11 (20%) | 0 (0%) | 0 (0%) | 1 (14%) | 0.674 |
| *SRSF2* | 3 (5%) | 1 (14%) | 0 (0%) | 1 (14%) | 0.359 |
| *DNMT3A* | 4 (7%) | 0 (0%) | 0 (0%) | 0 (0%) | 1.000 |
| *NF1* | 3 (5%) | 0 (0%) | 0 (0%) | 1 (14%) | 0.643 |
| *ZRSR2* | 4 (7%) | 0 (0%) | 0 (0%) | 0 (0%) | 1.000 |
| *RUNX1* | 3 (5%) | 0 (0%) | 0 (0%) | 0 (0%) | 1.000 |
| *BCOR* | 2 (4%) | 0 (0%) | 0 (0%) | 0 (0%) | 1.000 |
| *FBXW7* | 2 (4%) | 0 (0%) | 0 (0%) | 0 (0%) | 1.000 |
| *PDGFRA* | 2 (4%) | 0 (0%) | 0 (0%) | 0 (0%) | 1.000 |
| *PMS2* | 2 (4%) | 0 (0%) | 0 (0%) | 0 (0%) | 1.000 |
| *STAG2* | 1 (2%) | 0 (0%) | 1 (50%) | 0 (0%) | 0.091 |
| *TP53* | 2 (4%) | 0 (0%) | 0 (0%) | 0 (0%) | 1.000 |
|  |  |  |  |  |  |
| ***HMR, n (%)*** |  |  |  |  | 0.795 |
| 0 | 44 (79%) | 6 (86%) | 2 (100%) | 6 (86%) |  |
| 1 mutated gene | 9 (16%) | 1 (14%) | 0 (0%) | 0 (0%) |  |
| ≥2 mutated genes | 3 (5%) | 0 (0%) | 0 (0%) | 1 (14%) |  |
|  |  |  |  |  |  |
| No. of non-driver mutations, median (range) | 1 (0-8) | 0 (0-2) | 0.5 (0-1) | 0 (0-6) | 0.285 |

LDH, lactate dehydrogenase; TN, triple negative.

*Variables are defined as in Table 1.

^#^Categorical and continuous variables were analysed by Fisher exact and Kruskal-Wallis tests, respectively.

Significant *P* values (<0.05) are in bold.

**Table S4.** Correlations of non-driver gene mutations with clinical and laboratory findings in Chinese pre-PMF patients.

| **Gene*** | **Mutation** | ***n*** | **Male,**  ***n*** | **Female,**  ***n*** | ***P* value** | **Age,**  **median** | ***P* value** | **Hb, g/dL, median** | ***P* value** | **WBC, ×10^9^/L, median** | ***P* value** | **Plt, ×10^9^/L, median** | ***P* value** | **LDH, U/L, median** | ***P* value** |
| --- | --- | --- | --- | --- | --- | --- | --- | --- | --- | --- | --- | --- | --- | --- | --- |
| *TET2* | Yes | 13 | 12 | 1 | **0.024** | 70 | **0.027** | 12.7 | 0.889 | 29.4 | **0.009** | 690 | 0.141 | 316 | 0.543 |
|  | No | 59 | 33 | 26 |  | 62 |  | 12.7 |  | 14.4 |  | 858 |  | 308 |  |
|  |  |  |  |  |  |  |  |  |  |  |  |  |  |  |  |
| *ASXL1* | Yes | 12 | 10 | 2 | 0.190 | 70 | 0.051 | 12.6 | 0.118 | 19.0 | 0.051 | 896 | 0.397 | 304 | 0.832 |
|  | No | 60 | 35 | 25 |  | 65 |  | 13.3 |  | 13.9 |  | 735 |  | 286 |  |
|  |  |  |  |  |  |  |  |  |  |  |  |  |  |  |  |
| *SRSF2* | Yes | 5 | 5 | 0 | 0.150 | 71 | 0.198 | 9.5 | **0.046** | 38.5 | 0.161 | 620 | 0.731 | 364 | 0.287 |
|  | No | 67 | 40 | 27 |  | 65 |  | 13.2 |  | 14.1 |  | 744 |  | 283 |  |
|  |  |  |  |  |  |  |  |  |  |  |  |  |  |  |  |
| *DNMT3A* | Yes | 4 | 3 | 1 | 1.000 | 63 | 0.822 | 14.3 | 0.358 | 13.7 | 0.397 | 813 | 0.991 | 339 | 0.171 |
|  | No | 68 | 42 | 26 |  | 65 |  | 13.1 |  | 14.7 |  | 742 |  | 286 |  |
|  |  |  |  |  |  |  |  |  |  |  |  |  |  |  |  |
| *NF1* | Yes | 4 | 3 | 1 | 1.000 | 77 | 0.088 | 12.6 | 0.593 | 30.4 | **0.042** | 599 | 0.286 | 261 | 0.785 |
|  | No | 68 | 42 | 26 |  | 65 |  | 13.2 |  | 14.0 |  | 759 |  | 290 |  |
|  |  |  |  |  |  |  |  |  |  |  |  |  |  |  |  |
| *ZRSR2* | Yes | 4 | 4 | 0 | 0.290 | 76 | 0.051 | 11.0 | 0.121 | 12.0 | 0.577 | 586 | 0.411 | 230 | 0.083 |
|  | No | 68 | 41 | 27 |  | 65 |  | 13.2 |  | 14.5 |  | 761 |  | 296 |  |
|  |  |  |  |  |  |  |  |  |  |  |  |  |  |  |  |
| *RUNX1* | Yes | 3 | 3 | 0 | 0.287 | 49 | 0.433 | 11.4 | 0.227 | 17.3 | 0.238 | 846 | 0.853 | 325 | 0.400 |
|  | No | 69 | 42 | 27 |  | 65 |  | 13.1 |  | 14.1 |  | 739 |  | 288 |  |
|  |  |  |  |  |  |  |  |  |  |  |  |  |  |  |  |
| *BCOR* | Yes | 2 | 1 | 1 | 1.000 | 70 | 0.451 | 13.0 | 0.826 | 11.8 | 0.509 | 742 | 0.986 | 219 | 0.213 |
|  | No | 70 | 44 | 26 |  | 65 |  | 13.1 |  | 14.7 |  | 742 |  | 293 |  |
|  |  |  |  |  |  |  |  |  |  |  |  |  |  |  |  |
| *FBXW7* | Yes | 2 | 1 | 1 | 1.000 | 70 | 0.529 | 13.9 | 0.469 | 14.9 | 0.905 | 1119 | 0.213 | 311 | 0.728 |
|  | No | 70 | 44 | 26 |  | 65 |  | 13.1 |  | 14.4 |  | 738 |  | 290 |  |
|  |  |  |  |  |  |  |  |  |  |  |  |  |  |  |  |
| *PDGFRA* | Yes | 2 | 1 | 1 | 1.000 | 64 | 0.852 | 14.4 | 0.188 | 10.0 | 0.254 | 590 | 0.362 | 310 | 0.704 |
|  | No | 70 | 44 | 26 |  | 65 |  | 13.1 |  | 14.7 |  | 761 |  | 290 |  |
|  |  |  |  |  |  |  |  |  |  |  |  |  |  |  |  |
| *PMS2* | Yes | 2 | 1 | 1 | 1.000 | 73 | 0.329 | 13.9 | 0.469 | 14.4 | 1.000 | 795 | 1.000 | 232 | 0.329 |
|  | No | 70 | 44 | 26 |  | 65 |  | 13.1 |  | 14.4 |  | 742 |  | 290 |  |
|  |  |  |  |  |  |  |  |  |  |  |  |  |  |  |  |
| *STAG2* | Yes | 2 | 2 | 0 | 0.525 | 82 | **0.049** | 12.1 | 0.432 | 22.4 | 0.931 | 558 | 0.282 | 496 | 0.053 |
|  | No | 70 | 43 | 27 |  | 65 |  | 13.2 |  | 14.4 |  | 761 |  | 286 |  |
|  |  |  |  |  |  |  |  |  |  |  |  |  |  |  |  |
| *TP53* | Yes | 2 | 2 | 0 | 0.525 | 81 | 0.055 | 13.4 | 0.852 | 26.9 | 0.297 | 482 | 0.132 | 361 | 0.959 |
|  | No | 70 | 43 | 27 |  | 65 |  | 13.1 |  | 14.0 |  | 761 |  | 290 |  |

Hb, haemoglobin; WBC, white blood cells; Plt, platelets; LDH, lactate dehydrogenase.

^*^Only genes recurrently mutated (>1 patient) in the pre-PMF cases were studied.

Significant *P* values (<0.05) are in bold.

**Table S5.** Univariate and multivariate analysis of OS in Chinese pre-PMF patients.

|  |  | **Univariate** | | | **Multivariate**^#^ | | |
| --- | --- | --- | --- | --- | --- | --- | --- |
| **Variables** | ***n*** | **HR** | **95% CI** | ***P* value** | **HR** | **95% CI** | ***P* value** |
| Age>65 years | 33 | 4.05 | 1.46-11.28 | **0.007** | 3.32 | 1.03-10.76 | **0.045** |
| Male gender | 45 | 1.35 | 0.49-3.78 | 0.562 | . | . | . |
| Haemoglobin<10g/dL | 7 | 2.98 | 0.98-9.10 | 0.054 | . | . | . |
| Leukocytes>25×10^9^/L | 7 | 6.32 | 2.46-16.21 | **0.0001** | 3.57 | 1.25-10.18 | **0.017** |
| LDH>normal range | 56 | 0.68 | 0.26-1.81 | 0.442 | . | . | . |
| Constitutional symptoms | 12 | 1.98 | 0.75-5.27 | 0.169 | . | . | . |
| Splenomegaly | 22 | 1.30 | 0.51-3.33 | 0.580 | . | . | . |
|  |  |  |  |  |  |  |  |
| ***Driver types***^¶^ |  |  |  |  |  |  |  |
| *JAK2* unmutated | 16 | reference | | |  |  |  |
| *JAK2* V617F-low | 28 | 3.08 | 0.37-25.82 | 0.299 | . | . | . |
| *JAK2* V617F-high | 28 | 5.52 | 0.71-42.70 | 0.102 | . | . | . |
|  |  |  |  |  |  |  |  |
| ***Non-driver mutations***^*^ |  |  |  |  |  |  |  |
| *TET2* | 13 | 3.79 | 1.49-9.61 | **0.005** | . | . | . |
| *DNMT3A* | 4 | 0.92 | 0.12-6.96 | 0.932 | . | . | . |
| *NF1* | 4 | 3.38 | 0.97-11.83 | 0.057 | . | . | . |
| *ZRSR2* | 4 | 0.85 | 0.11-6.47 | 0.879 | . | . | . |
| *RUNX1* | 3 | 3.97 | 0.90-17.49 | 0.069 | 8.03 | 1.55-41.62 | **0.013** |
| *BCOR* | 2 | 4.93 | 0.61-39.68 | 0.133 | . | . | . |
| *PMS2* | 2 | 5.54 | 0.67-46.02 | 0.113 | . | . | . |
| *STAG2* | 2 | 3.06 | 0.40-23.46 | 0.283 | . | . | . |
| *TP53* | 2 | 15.99 | 3.07-83.43 | **0.001** | 8.18 | 1.43-46.78 | **0.018** |
|  |  |  |  |  |  |  |  |
| **HMR** |  |  |  |  |  |  |  |
| 0 | 58 | reference | | |  |  |  |
| 1 mutated gene | 10 | 1.69 | 0.54-5.30 | 0.367 | . | . | . |
| ≥2 mutated genes | 4 | 4.61 | 1.29-16.44 | **0.019** | . | . | . |

LDH, lactate dehydrogenase; HR, hazard ratio; 95% CI, 95% confidence interval.

^¶^Driver types were stratified according to the *JAK2* V617F status. Low and high indicate mutant burden below and above the median, respectively.

^*^Only genes recurrently mutated (>1 patient) in the pre-PMF cases were studied. *FBXW7* and *PDGFRA* mutations were not evaluated because all the mutated cases were censored.

^#^Variables with *P*<0.1 in univariate analysis were included in the multivariate analysis. Same results were obtained when the forward or backward stepwise selection was used.

Significant *P* values (<0.05) are in bold.

**Figure S1. Risk stratification of pre-PMF patients according to other prognostic scoring systems.** (A) Risk stratification based on DIPSS+. (B) Risk stratification based on MIPSS70+. (C) Risk stratification based on GIPSS. Patients with low-risk (*n*=3) were all censored and combined with intermediate-1-risk cases for analysis. (D) Risk stratification based on MIPSS70+ version 2.0. Patients with very low-risk (*n*=3) were all censored and combined with low-risk cases for analysis. In all panels, only patients with cytogenetic information (*n*=50) were studied. Kaplan-Meier survival curves were compared by log-rank test. Int-1 and Int-2 represent intermediate-1 and intermediate-2, respectively.
